# Supplementary material for: Causal Relationship between Aging and Anorexia Nervosa: A White-Matter-Microstructure-Mediated Mendelian Randomization Analysis
Source: Biomedicines. 2024 Aug 16;12(8):1874. doi: 10.3390/biomedicines12081874 (PMC11351342; doi:10.3390/biomedicines12081874)
Supplement: Supplementary file 1 [file biomedicines-12-01874-s001.zip › Supplementary Figure/Figure S4/Singe SNP plot of MR analysis for TL to ubm-b-1566.pdf]

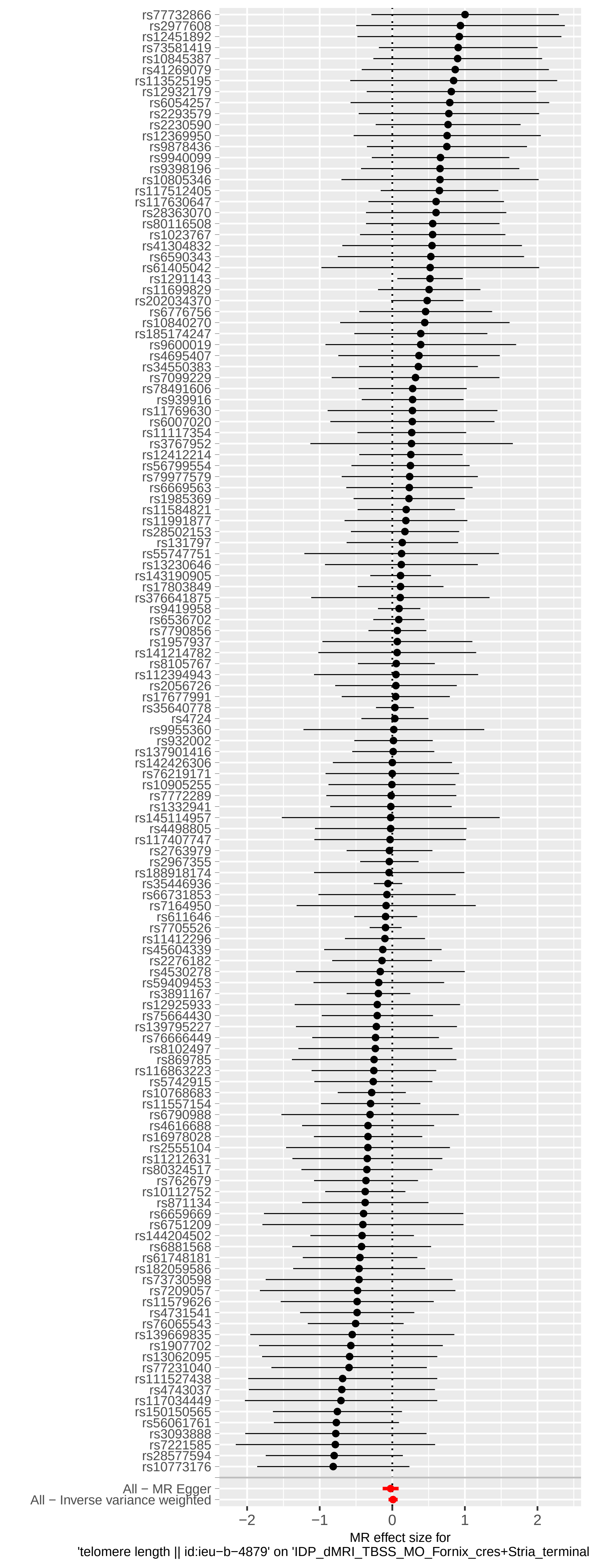
rs77732866
rs2977608
rs12451892
rs73581419
rs10845387
rs41269079
rs113525195
rs12932179
rs6054257
rs2293579
rs2230590
rs12369950
rs9878436
rs9940099
rs9398196
rs10805346
rs117512405
rs117630647
rs28363070
rs80116508
rs1023767
rs41304832
rs6590343
rs61405042
rs1291143
rs11699829
rs202034370
rs6776756
rs10840270
rs185174247
rs9600019
rs4695407
rs34550383
rs7099229
rs78491606
rs939916
rs11769630
rs6007020
rs11117354
rs3767952
rs12412214
rs56799554
rs79977579
rs6669563
rs1985369
rs11584821
rs11991877
rs28502153
rs131797
rs55747751
rs13230646
rs143190905
rs17803849
rs376641875
rs9419958
rs6536702
rs7790856
rs1957937
rs141214782
rs8105767
rs112394943
rs2056726
rs17677991
rs35640778
rs4724
rs9955360
rs932002
rs137901416
rs142426306
rs76219171
rs10905255
rs7772289
rs1332941
rs145114957
rs4498805
rs117407747
rs2763979
rs2967355
rs188918174
rs35446936
rs66731853
rs7164950
rs611646
rs7705526
rs11412296
rs45604339
rs2276182
rs4530278
rs59409453
rs3891167
rs12925933
rs75664430
rs139795227
rs76666449
rs8102497
rs869785
rs116863223
rs5742915
rs10768683
rs11557154
rs6790988
rs4616688
rs16978028
rs2555104
rs11212631
rs80324517
rs762679
rs10112752
rs871134
rs6659669
rs6751209
rs144204502
rs6881568
rs61748181
rs182059586
rs73730598
rs7209057
rs11579626
rs4731541
rs76065543
rs139669835
rs1907702
rs13062095
rs77231040
rs111527438
rs4743037
rs117034449
rs150150565
rs56061761
rs3093888
rs7221585
rs28577594
rs10773176
All − MR Egger
All − Inverse variance weighted
−2
−1
0
1
2
MR effect size for
'telomere length || id:ieu−b−4879' on 'IDP_dMRI_TBSS_MO_Fornix_cres+Stria_terminal
